# Identification of an ATP-Binding Cassette Transporter Implicated in Aluminum Tolerance in Wild Soybean (*Glycine soja*)

**DOI:** 10.3390/ijms222413264

**Published:** 2021-12-09

**Authors:** Ke Wen, Huanting Pan, Xingang Li, Rong Huang, Qibin Ma, Hai Nian

**Affiliations:** 1 The State Key Laboratory for Conservation and Utilization of Subtropical Agro-Bioresources, South China Agricultural University, Guangzhou 510642, China; wenqicool@126.com (K.W.); tlz100118@126.com (H.P.); lxg18218673393@163.com (X.L.); 15180541095@163.com (R.H.); maqibin@scau.edu.cn (Q.M.); 2 The Key Laboratory of Plant Molecular Breeding of Guangdong Province, College of Agriculture, South China Agricultural University, Guangzhou 510642, China; 3 The National Engineering Research Center of Plant Space Breeding, South China Agricultural University, Guangzhou 510642, China; 4 The Guangdong Subcenter of the National Center for Soybean Improvement, College of Agriculture, South China Agricultural University, Guangzhou 510642, China; 5 Zengcheng Teaching and Research Bases, South China Agricultural University, Guangzhou 510642, China

**Keywords:** *GsABCI1*, ABC transporter, aluminum tolerance, wild soybean, overexpression

## Abstract

The toxicity of aluminum (Al) in acidic soil limits global crop yield. The ATP-binding cassette (ABC) transporter-like gene superfamily has functions and structures related to transportation, so it responds to aluminum stress in plants. In this study, one half-size ABC transporter gene was isolated from wild soybeans (*Glycine soja*) and designated *GsABCI1*. By real-time qPCR, *GsABCI1* was identified as not specifically expressed in tissues. Phenotype identification of the overexpressed transgenic lines showed increased tolerance to aluminum. Furthermore, *GsABCI1* transgenic plants exhibited some resistance to aluminum treatment by ion translocation or changing root components. This work on the *GsABCI1* identified the molecular function, which provided useful information for understanding the gene function of the ABC family and the development of new aluminum-tolerant soybean germplasm.

## 1. Introduction

Acidic soils account for more than 30% of land area worldwide, particularly in the tropics and subtropics [1,2,3,4]. In soils with pH below 5, insoluble aluminum is mainly released into the soil in the form of soluble Al^3+^, where it will promptly inhibit the elongation of plants and the absorption of water and nutrient by roots, eventually leading to the loss and quality decline of crop products [5,6,7]. In fact, in an acidic soil environment, the toxicity of aluminum has become the main abiotic stress and limiting factor for crop production [8,9,10]. In order to resist the toxicity of aluminum, most plant species have adopted mechanisms in long-term evolution, namely, external exclusion and internal tolerance mechanisms. Exclusion mechanisms have a range of processes in plants, including the secretion of carboxylic acids (citrate, oxalate, malate), conservation of the cell wall, secretion of inorganic phosphorus, transmembrane efflux, and regulation of rhythmic pH variation [11,12,13,14,15,16,17]. The internal mechanism is to sequester or isolate Al to vacuoles and other areas insensitive to aluminum [18,19,20,21,22].

At present, aluminum tolerance gene families have been identified in plant species. The ATP-binding cassette (ABC) transporter protein superfamily comprises diverse, complex, and ubiquitous proteins [23]. The *Arabidopsis thaliana*, rice, and soybean genomes contain an estimated 131, 121, and 261 members of the ABC family, respectively, and these proteins are divided into 8 basic subfamilies (ABCA-ABCG and ABCI) based on the domain organization and homologous relationship [24,25,26]. ABC transporters are a family of membrane-bound proteins that mediate transport across biofilms by hydrolyzing ATP. They contain a conservative cytoplasmic domain, called the nucleotide-binding domain (NBD) (also known as an ATP-binding cassette) [26]. In addition to this domain, ABC protein also includes one or two hydrophobic transmembrane domains (TMDs). Recently, studies have suggested that the ABC transporter proteins in plants not only participate in the transport of alkaloids, steroids, phenols, lipids, metal ions, hormones, salts, organic acids, and xenobiotics but also contribute to the modulation of ion channels and plant–pathogen interactions [27,28,29,30,31,32]. In some studies, several members of the ABC transporter superfamily were identified as being involved in enhancing resistance to aluminum toxicity. For instance, in rice, aluminum toxin-sensitive proteins *OsSTAR1* and *OsSTAR2* were identified, which are responsible for UDP-glucose efflux [33]. It has been speculated that the transported UDP-glucose is the precursor substance of hemicellulose and pectin of cell wall components, thereby exerting the modification function of the cell wall, limiting the accumulation of aluminum, and reducing the toxicity of aluminum [33,34,35]. Similarly, *FeSTAR1*, *FeSTAR2*, and *SbSTAR1* have similar functions in buckwheat and sweet sorghum [36,37]. Larsen discovered three aluminum-sensitive proteins encoded by an ABC transporter gene (*ALS3*), which was necessary for the redistribution of Al^3+^ from highly sensitive tissues [38]. Its homologous genes *FeALS3* and *GmALS3* were also identified in buckwheat and soybean [39,40]. Moreover, some ABC transporter genes, such as *HvABCB25*, *CcABCG7*, *FeALS1.1*, *FeALS1.2*, and *OsALS1*, were also isolated, conferring aluminum tolerance in wild barley, pigeon pea, buckwheat, and rice, separately [41,42,43]. Among them, *OsALS1* encodes an aluminum transporter protein, which is supposed to work cooperatively with *OsNrat1* and participate in aluminum translocation [43,44].

Soybean (*Glycine max*) is one of the most important oil and protein crops in the world, but its genetic diversity has decreased in the process of artificial domestication and breeding selection from wild soybean to cultivated soybean [45]. Wild soybean (*G. soja*) is believed to have a broader genetic basis and a stronger tolerance for abiotic stress, which provides opportunities to identify the potential genes related to Al resistance [46]. In the current study, a half-size ABC transporter gene was isolated from the BW69 line (Al-resistant) of wild soybean (*G. soja*), named *GsABCI1*, and the response of the gene expression pattern to aluminum treatment was detected by qPCR. The function of *GsABCI1* was identified and subjected to subcellular localization and phenotypic analysis of root components as well as transgenic overexpression lines from *A. thaliana* and *G. max*. We hypothesized that *GsABCI1* could improve the aluminum tolerance of wild soybean through aluminum translocation or alteration of root composition.

## 2. Results

### 2.1. GsABCI1 Encodes an ATP-Binding Cassette Transporter Protein

**Figure 1:**
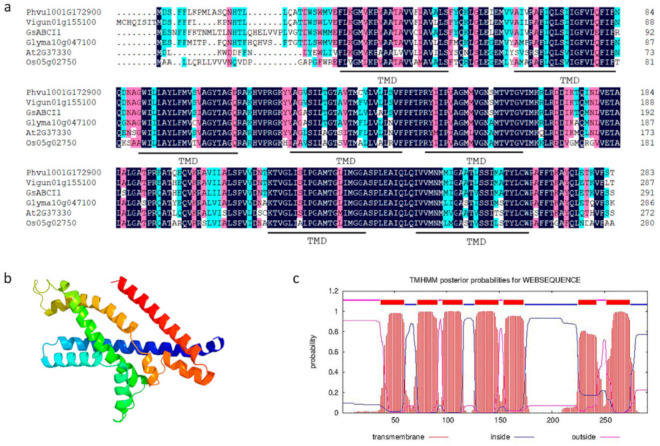
(**a**) Multiple sequence alignment and protein structure analysis of the *GsABCI1* and ABC domain of other species. The line below the sequence indicates the predicted conserved trans-membrane domains (TMDs). The black part indicates the amino acids shared between the sequences. *Phvul.001G172900*: *Phaseolus vulgaris*; *Vigun.01g155100*: *Vigna unguiculata*; *Glyma.10g175800*: *Glycine max*; *At2g37330*: *Arabidopsis thaliana*; *Os05g02750*: *Oryza sativa*. (**b**) Prediction of the *GsABCI1* protein structure model. One color represents one protein fold. (**c**) Prediction of the transmembrane domain of *GsABCI1* protein. Red line: transmembrane structure, blue line: inside the membrane, and purple line: outside the membrane.

**Figure 2:**
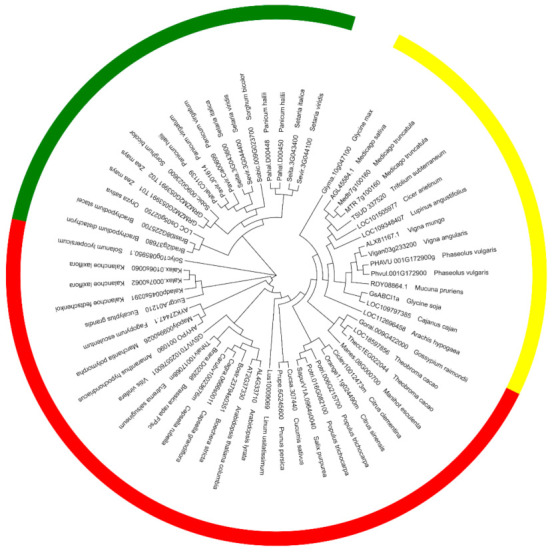
Phylogenetic analysis of the *GsABCI1* protein and other ABC transporter proteins. The different colors represent the species type. Yellow, red, and green represent legume, dicotyledons (except the legume), or monocotyledons.

Based on the gene expression profile of aluminum-resistant wild soybean (unpublished data), an aluminum-induced gene encoding ABC transporter protein was cloned. A BLAST search using the ABC protein sequence identified two unique homologues, *Glyma03g175800 (GmABCI1)* and *Glyma10g047100 (GmABCI13)*, in the soybean genome of Williams82. By sequence alignment, we found that only two base sequences located at 567 and 720 were mutated in *Glyma03g175800*, Therefore, we named the isolated gene *GsABCI1* according to the Human Genome Organization (HUGO) scheme and previous studies [25]. The full-length genome sequence of *GsABCI1* included 3 exons and 2 introns with a cDNA of 1163 bp in length, including a 678 bp open reading frame (ORF), and encodes a peptide of 291 amino acids (Additional file S1: Table S1). At the amino acid level, *GsABCI1* shows 76% and 62% identity with *AtALS3* (*At2g37330*, *A. thaliana*) and *OsSTAR2* (*Loc_Os01g0674700*, *Oryza sativa*), respectively. *Phvul.001G172900* from *Phaseolus vulgaris* shared 92.8% identity with *GsABCI1* and is the closest homologue of *Leguminosae* sp. Prediction using TMHMM (Server v. 2.0) and Protein Homology/analogY Recognition Engine v2.0 showed that *GsABCI1* is a permease protein that has seven predicted transmembrane domains (TMDs, 37–59, 72–91, 96–115, 127–149, 154–173, 225–242, 252–274) without nucleotide-binding domains (NBD), and therefore was classified as a half-size ABC transporter. (Figure 1c). According to the phylogenetic analysis, *GsABCI1* homologues exist in different species including dicotyledons and monocotyledons. *GsABCI1* is clustered with legume species, so it is speculated that the genes of the ABC family are conservative to some extent in a wide range of legume species (Figure 2).

### 2.2. GsABCI1 Expression Responses to Aluminum Stress in Wild Soybean (Glycine soja)

**Figure 3:**
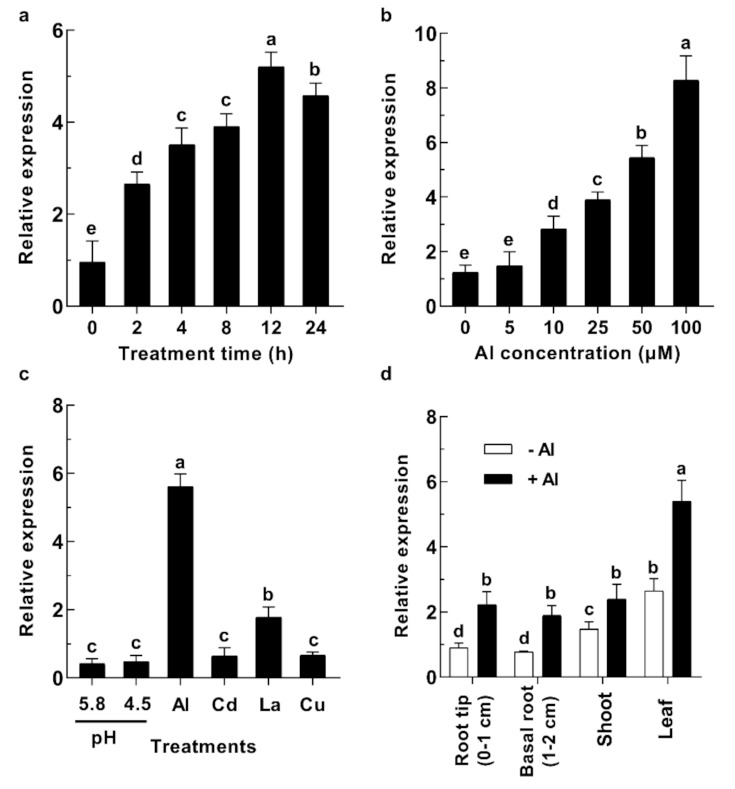
The detection of the *GsABCI1* expression pattern. (**a**) Time-dependent expression pattern of *GsABCI1*. Plants were treated with a solution containing 50 μM AlCl_3_ for 0, 2, 4, 8, 12, and 24 h. Root tips (0–1 cm) were collected for qPCR after different time treatments. (**b**) Dose-dependent expression pattern of *GsABCI1*. Plants were exposed to a solution containing 0, 5, 10, 25, 50, or 100 μM AlCl_3_ for 8 h. Root tips (0–1 cm) were collected for qPCR after 8 h in different Al treatments. (**c**) The *GsABCI1* expression of response to metals and different pH values. Seedling plants were grown under different pH (pH 4.5, or 5.8) conditions or in solutions (pH 4.5) containing 50 μM AlCl_3_, 23 μM CdCl_3_, 10 μM LaCl_3_, or 0.5 μM CuCl_2_ for 8 h. Root tips (0–1 cm) were collected for qPCR after 8 h in different treatments. (**d**) Tissue-specific expression pattern of *GsABCI1*. Seedling plants were cultivated in a solution with 0 or 50 μM AlCl_3_ for 12 h. Root tips (0–1 cm), Basal roots (1–2 cm), shoots, and leaves were collected for qPCR after treatment. The expression levels of all candidate genes were determined by using the 2^−ΔΔct^ method. The data are the means ± SDs (*n* = 3). The different letters indicate significant differences at *p* < 0.05 as determined by Student’s *t*-test.

The time-response experiment revealed that *GsABCI1* was expressed in the root within 2 h of Al treatment and that the expression level increased significantly with prolonged treatment time. However, the expression level began to decline after 12 h of treatment (Figure 3a). Furthermore, as shown in Figure 3b, the expression of *GsABCI1* in the roots was prominently increased with increasing Al concentration after eight hours of exposure. In the experiment examining the response to metals and different pH, the expression of *GsABCI1* was induced by Al and lanthanum (La) but not by copper (Cu) or cadmium (Cd), and the induced expression was dramatically lower with La than Al. Between the two pH values, the expression of *GsABCI1* was not increased with pH 4.5 (Figure 3c). In the tissue-specific expression experiment, the expression of *GsABCI1* was concentrated in the root, shoot, and leaf with or without Al exposure, and the expression in leaves was higher than that in roots (Figure 3d).

### 2.3. Generation and Detection of GsABCI1 Transgenic Lines

To identify the function of *GsABCI1*, the vector containing a 35S strong promoter original was constructed, and several lines of the transgenic plant were obtained by staining and cotyledon node transformation, respectively. The transgenic T_1_ *A. thaliana* and *G. max* were transformed and confirmed by PCR identification with a specific primer for the amplification of the glufosinate-resistant genes (Additional file S2: Table S2). Combined with herbicide identification, the results demonstrated that the *GsABCI1* was integrated into the genomes of recipient plants (Additional file S3: Figure S1). Three overexpression transgenic lines of each soybean and *A. thaliana* with high gene expression in the T_3_ generation as determined by qPCR were selected to study the Al tolerance phenotype of transgenic *GsABCI1* plants.

### 2.4. Overexpression of GsABCI1 Enhances Phenotypic Tolerance to Aluminum

**Figure 4:**
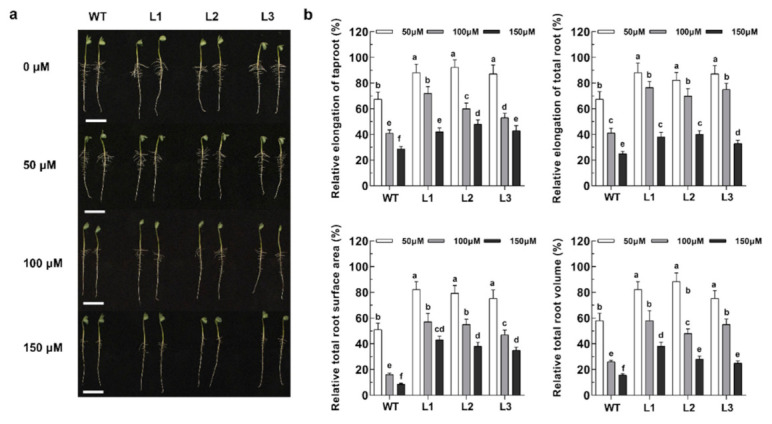
Phenotypic detections of the *GsABCI1* transgenic soybean lines. (**a**) Morphological analysis of the roots of the WT and three transgenic lines (L1, L2, L3). (**b**) Analysis of relative taproot elongation, relative total root elongation, relative total root surface area, and relative total root volume in the WT and three transgenic lines (L1, L2, L3). Soybean seedlings with consistent growth were identified and transferred into a 0.5 μM CaCl_2_ hydroponic solution (pH 4.5) containing 0, 50, 100, or 150 μM AlCl_3_ for 12 h. Photograph taken, scanned, and acquired data through Image J. Bar = 5 cm. The data are the means ± SDs (*n* = 16). The different letters indicate significant differences at *p* < 0.05 as determined by Student’s *t*-test.

To compare the growth phenotypes of the wild type (WT) and three transgenic lines (L1, L2, L3) at physiologically relevant concentrations of Al, an Al dose–response analysis in solution culture was performed. As shown in Figure 4a, the root elongation of both the WT and transgenic soybean lines was inhibited to some extent by various concentrations of Al and the higher the concentration of Al was, the greater the suppression effect. However, the three transgenic lines were significantly less inhibited than the WT. For instance, when the Al concentration was 100 μM, the taproot elongation of WT was inhibited by 58%, while that of L1, L2, and L3 were inhibited by 28%, 44%, and 46%, respectively (Figure 4b). The auxiliary data describing the relative total area, relative total root length, and relative total volume illustrate that the root growth of the transgenic lines was less affected by the toxic compound than that of the WT under Al stress (Figure 4). In the presence of Al, both the WT and transgenic *A. thaliana* lines were severely inhibited under 150 μM Al treatment, but the relative root elongation of the transgenic *A. thaliana* was significantly higher than that of the WT under Al treatment (Additional file S4: Figure S2). Those results indicate that the overexpression of *GsABCI1* in transgenic *A. thaliana* or *G. max* confers increased resistance to aluminum toxicity.

### 2.5. GsABCI1 Involves the Translocation of Aluminum in Roots

**Figure 5:**
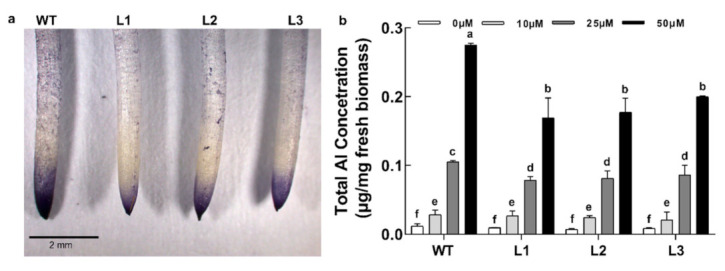
Analysis of Al accumulation patterns. (**a**) Al-treated (25 μM AlCl_3_ for 24 h) roots of WT and transgenic soybean lines were stained with hematoxylin, washed, and photographed. Bar = 2 mm. (**b**) The total Al accumulation in the roots. Soybean seedlings with consistent growth were transferred into a 0.5 μM CaCl_2_ hydroponic solution (pH 4.5) containing 0, 10, 25, or 50 μM AlCl_3_ for 24 h. The Al of the roots (0–6 cm) was extracted by 2N HCl and determined. The data are the means ± SDs (*n* = 12). The different letters indicate significant differences at *p* < 0.05 as determined by Student’s *t*-test.

The roots of the soybean were stained with the aluminum indicator dye hematoxylin, and the total aluminum accumulation was analyzed by inductively coupled plasma mass spectrometry (ICP-MS). The diffusion pattern of surface-bound Al^3+^ in the transgenic lines was substantial and extended from the apical region to the mature region. The WT root had strong staining in a local area near the root tip (Figure 5a), which is the area where plants are sensitive to Al toxicity. Additionally, the ICP-MS quantification of Al accumulation showed slight differences in the total Al concentration in the roots (0–2 cm) of the WT and transgenic lines (Figure 5b). Generally, under high Al concentrations (25 or 50 μM), the Al^3+^ accumulation of the transgenic lines was less than that of the WT strain, but the difference was not significant under the low concentration (10 μM) (Figure 5b).

### 2.6. Root Component Changes of GsABCI1 Transgenic Lines under Aluminum Stress

**Figure 6:**
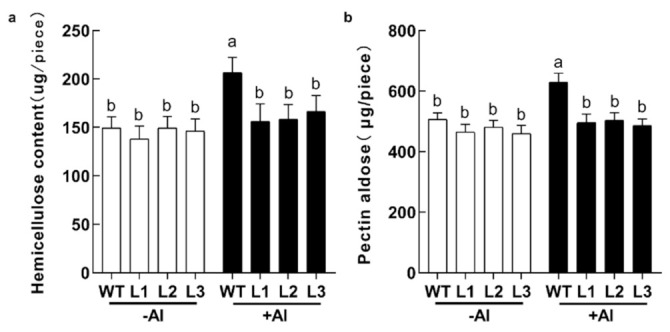
Root component of the *GsABCI1* transgenic soybean lines. (**a**) Hemicellulose content in the root cell wall of the wild type and three transgenic lines. (**b**) Hemicellulose or pectin aldose was extracted from the root cell wall of plants with or without 50 μM aluminum treatment. The data are the means ± SDs (*n* = 12). The different letters indicate significant differences at *p* < 0.05 as determined by Student’s *t*-test.

The soybean root cell wall has been recognized as the primary target of aluminum toxicity, with hemicellulos and pectin being the major constituents of the cell wall. The homologous gene of *GsABCI1* was identified to be potentially involved in the transport of UDP-glucose and thus alter cell wall composition. Therefore, we analyzed whether the aluminum resistance of the transgenic lines was associated with hemicellulose and pectin aldose metabolism. As shown in Figure 6, the hemicellulose and pectin aldose contents of the *GsABCI1* transgenic line were significantly lower than those of the wild type under aluminum treatment.

### 2.7. GsABCI1 Subcellular Localization in the Plasma Membrane

**Figure 7:**
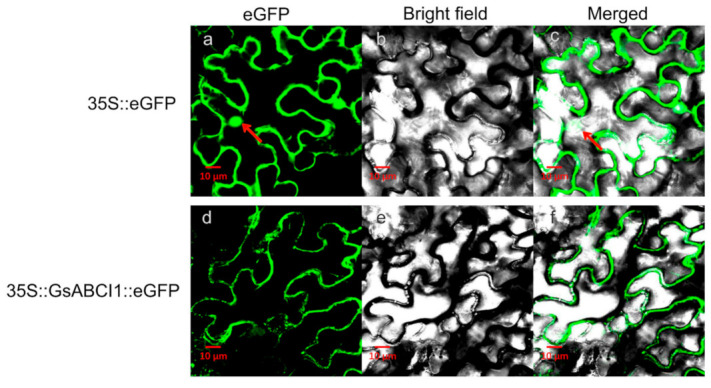
Subcellular localization of *GsABCI1* in Nicotiana benthamiana cells. (**a**–**c**) Localization of 35S::eGFP in N. benthamiana leaf cells. (**d**–**f**) Localization of 35S:GsABCI1::eGFP in N. benthamiana leaf cells. Fluorescence signs in the epidermal cells were analyzed by confocal microscopy. The specific vector was transformed into N. benthamiana leaf by an infiltration method. (**a**,**c**) The arrows indicate the position of the nucleus.

To determine the subcellular localization of *GsABCI1*, the GsABCI1-GFP fusion protein was transiently expressed in epidermal cells of *Nicotiana benthamiana*. As shown in Figure 7a–c, GFP emitted green fluorescence throughout the whole cell. In contrast, the GsABCI1-GFP fusion protein had no signal in the nucleus but had a strong green fluorescent signal in the cell membrane (Figure 7d–f). In general, these results indicate that *GsABCI1* protein is localized to the plasma membrane as predicted by SMART (http://smart.embl-heidelberg.de/ (accessed on 6 June 2021)).

## 3. Discussion

The toxicity of aluminum has a far-reaching impact on agriculture all over the world, which severely limits crop yields by inhibiting root growth and nutrient absorption [6,47,48,49]. At present, the function and mechanism of anti-Al-related genes in some plants have been identified, including *AtALMT1*, *TaALMT1*, *GmALMT1*, *AtMATE*, *VuAAE3*, *TaMATE1*, *OsFRDL4*, *OsSTAR1*, *OsSTAR2*, *AtMGT1*, *OsNrat1*, and *AtSTOP1*, which can provide basic data for elucidating the physiology and genetics of aluminum tolerance mechanisms [33,47,50,51,52,53,54,55,56,57,58]. It has been found that the ABC transporter gene superfamily has the function of material transport related to plant responses to abiotic stresses, and plays a key role in plant physiology and development [25]. The ABCI subfamily is a unique ABC family in plants, which does not exist in animals [59]. It has been identified that gene members of many species are related to aluminum tolerance. *OsSTAR1* and *OsSTAR2* are members of the ABCI subfamily, which is one of the most important mechanisms of aluminum toxicity in rice [33]. In dicotyledonous plants Arabidopsis *thaliana AtABCI16* and *AtABCI17*, the corresponding aluminum toxicity was also identified [59]. Larsen discovered Al-sensitive mutants and isolated the gene *ALS3*(*At2g37330*), which caused this phenomenon of Al insensitivity [38]. Soybean is one of the most widely cultivated crops in the world, and it is also an important source of human protein, oil, and animal feed. A few days ago, it was identified that the ABCI subfamily gene *GmABCI13*(*Glyma.10g47100*) was highly expressed in the soybean roots under aluminum stress, and it was also considered as the orthologous gene of *ALS3*, which indicated that the ABCI subfamily had extensive aluminum tolerance in plants [40]. In this study, we identified the ABCI subfamily gene *GsABCI1* in wild soybean (*G. soja*) as being related to aluminum tolerance and speculated that it might be involved in the transportation of cell wall structural components.

By sequence alignment, *GsABCI1* showed a high degree of homology with the already identified ABCI subfamily genes, especially with *GmABCI13* (Figure 1a). *GsABCI1,* which contains the transmembrane domains of the ABCI transporter, the conservative transmembrane domain, is similar to its homologues and is assumed to have a similar function (Figure 1). The phylogenetic analysis of *GsABCI1* protein and other ABC transporter shows that the *GsABCI1* protein sequence was conserved among different species, especially among dicotyledons. It encodes a previously uncharacterized half-type ABC transporter system permease protein, which consists of seven transmembrane domains and does not contain an ATP-binding domain, which means that it must interact with other proteins to provide the energy needed to drive substrate movement [38,43,60]. This seems to be similar to the interaction between *OsSTAR1* and *OsSTAR2* in rice, and further research is needed [33].

While similar in structure to homologous ABC family genes, *GsABCI1* still has its unique functions. The expression pattern of *GsABCI1* shares many similarities with that of the dicot gene, *At2g37330*, but is different from that of the monocot gene, *OsSTAR2* [33,49]. The expression of *GsABCI1* is not limited to the roots like *OsSTAR2* but is expressed in various tissues of the plant, especially in the stems and leaves (Figure 3d). After 12 h of aluminum stress, the expression level of *GsABCI1* reached its maximum and the peak of the expression level was later than that of rice *OsSTAR2*. It was speculated that Al might first act on the binding of other substances or transcription factors in soybean root cells, and then induce the expression of *GsABCI1* (Figure 3a) [61,62]. Furthermore, we speculated that the transmembrane domain of *GsABCI1* may be the pathway of trivalent metal transport, because the expression of *GsABCI1* is specifically increased by exposure of Al^3+^ and La^3+^ but not Cd^2+^ or Cu^2+^ (Figure 3c). The subcellular cells of the *GsABCI1* protein were localized to the plasma membrane (Figure 7). Therefore, it is speculated that *GsABCI1* may be involved in the transmembrane transport of some substances.

In order to further verify the role of *GsABCI1* in aluminum resistance, *GsABCI1* was transformed into cultivated soybean and A. *thaliana*. Compared with the treatment control, with the increase of the aluminum concentration, aluminum stress inhibited the elongation of the main root of wild-type and transgenic soybean or A. *thaliana* (Figure 4, Additional file S4: Figure S2). However, the relative root elongation and other root morphology data of transgenic lines were significantly higher than those of the wild type. Consistent with the speculation, the overexpression of *GsABCI1* enhanced the tolerance of A. *thaliana* or soybean to aluminum. Similar abiotic stress phenotypes were also studied in other ABC genes of other crops. For example, *SbSTAR1* enhanced the aluminum tolerance of transgenic *Arabidopsis thaliana* by regulating hemicellulose [37]. In this study, the contents of hemicellulose and pectin aldose in *GsABCI1* transgenic soybean lines and wild-type roots were also determined to study the response mechanism of soybean to aluminum stress, and the results were similar to previous studies [17,33,37,62,63,64,65,66]. We speculated that *GsABCI1* transgenic soybean may enhance the tolerance of plants to aluminum stress by mediating the changes of hemicellulose and pectin aldose, but it does not change under the condition of aluminum treatment, which seems to be not directly mediated (Figure 6). The measurement of hematoxylin staining and aluminum accumulation showed that compared with the WT strain, the total aluminum accumulation in the root tip of transgenic strain decreased, while the total aluminum accumulation in the distal tip slightly decreased (Figure 5). Other researchers suggested that both *ALS3* and *ALS1* may be involved in the mechanism of aluminum movement in insensitive tissues or subcellular structures [38,60]. Therefore, it is speculated that *GsABCI1* also mediates the loading or unloading of aluminum and its transportation from the root tip to the root elongation area or even to the aluminum-insensitive leaf area, thus enhancing the tolerance of plants to aluminum toxicity [67]. We speculated two possible mechanisms of *GsABCI1*-mediated aluminum tolerance, which may need further exploration. Consequently, further study on the function of the *GsABCI1* gene will enrich our understanding of the mechanism of aluminum tolerance in plants.

## 4. Materials and Methods

### 4.1. Plant Materials and Growing Conditions

The seeds of wild soybean BW69 were obtained from the Guangdong Sub-Center of the National Center for Soybean Improvement (Guangzhou, China). To break dormancy and enhance germination, sterile seeds were treated with a sulfuric acid solution for 15 min, washed thoroughly with sterile double-distilled water (ddH_2_O) five times, and germinated in soil mixed with quartz sand and vermiculite (1:1) for two days. Uniformly germinated seeds were transferred to a hydroponic system (pH 5.8) containing 1/4 Hoagland’s solution at 25 °C, 60% relative humidity (RH), 550 µmol m^−2^ s^−1^ light intensity, and 16 h/8 h (light/dark) photoperiod [68,69]. The nutrient solution was renewed every two days. After disinfection and vernalization, A. thaliana ecotype Columbia (Col-0) and transgenic lines were grown in a greenhouse under the following conditions: 21–23 °C; 60% relative humidity; 100 μmol m^−2^ s^−1^ light intensity; and 16 h/8 h (light/dark) photoperiod.

### 4.2. Isolation of the GsABCI1 Gene from Wild Soybean and Vector Construction

To isolate the *GsABCI1* gene, total RNA was extracted from the roots of wild soybeans BW69 by TRNzol Reagent (Tiangen Biotech, Beijing, China). The conversion to cDNA was performed by a PrimeScript RT Reagent Kit with gDNA eraser (TaKaRa, Shiga, Japan) according to the manufacturer’s procedures. Based on the previous analysis of acidic aluminum tolerance-related gene expression profiles (unpublished data), *GsABCI1* was amplified by a specific primer (Additional file S2: Table S2). The PCR product was inserted into the pLB-simple vector and confirmed by sequencing. The above method referred to a previously described method [70].

For the overexpression of *GsABCI1* in soybean and A. *thaliana*, the coding region of *GsABCI1* was amplified using gene-specific primers with the *XbaI* site and *SacI* site (Additional file S2: Table S2). *GsABCI1* was then cloned into the *pTF101.1* binary vector with the phosphinothricin acetyltransferase (bar) resistance gene (encoding phosphinothricin N-acetyltransferase, PAT, conferring resistance to glufosinate herbicide) as the plant selection marker, which was driven by a modified CaMV 35S promoter [71].

### 4.3. Generation of GsABCI1 Transgenic Lines

The recombinant vector *pTF101.1-GsABCI1* was transformed into agrobacterium tumefaciens strain GV3101 (for A. *thaliana*) or EHA101 (for soybean) by electroporation (Gene Pulser Xcell™ Electroporation Systems, Hercules, California, US) and confirmed by PCR [72,73]. A. *thaliana* ecotype Columbia (Col-0) was used for transformation by the floral dip method [74]. The nationally approved soybean cultivar (Ministry of Agriculture and Rural Affairs, Beijing, China), Huachun6, was used as the recipient for Agrobacterium-mediated genetic transformation, as previously described with slight modification [75,76,77]. In short, mature soybean seeds without spots were plated on Petri dishes and sterilized. After exposing the sample to airflow for two hours to remove the Cl_2_, the disinfected soybean seeds were sown in the germination medium (GM) for five days. The primary and shoot seed coats were removed from the germinated seeds, and the cotyledonary nodes were cut approximately 10 times with a scalpel. Afterward, the explants were infected for 30 min by immersion in a liquid with the Agrobacterium suspension and placed on cocultivation medium (CM) in a darkroom for three days. After cocultivation, the explants were washed and inserted into sprout-induced medium (SIM) with Timentin (Realtimes, Beijing, China) at 30–45° angles to induce shoots for two weeks under. Then, the hypocotyl of the explants was cut again and cultured on sprout-induced medium for another two weeks. The numerous induced shoots were transferred to sprout elongation medium (SEM) with Timentin for 2–8 weeks, and the new sprout elongation medium was changed every two weeks. The explants that grew larger than 3 cm were dipped in an indole-3-butyric acid (IBA) solution for 1–4 min and transferred to root spread medium (RSM) with Timentin for two weeks. The seedlings were transplanted to sterile soil, and the seeds were harvested in the culture chamber. All medium components used for genetic transformation are shown in the table (Additional file S5: Table S3).

### 4.4. Detection of GsABCI1 Transgenic Lines

T_0_ transgenic soybean plants were tested by phosphinothricin following the manufacturer’s instructions. In the early flowering period of soybean, herbicide (Liberty^®^, Bayer, Monheim am Rhein, Germany) was applied at the same position as the third compound leaf of the transgenic soybean at a concentration of 135 mg L-1, and the soybean leaf condition was observed three days after application. Similarly, T_0_ transgenic A. *thaliana* plants were sprayed with herbicide (Liberty^®^, Bayer, Pittsburgh, PA, USA) for identification. In addition, for molecular identification, total DNA was extracted from the leaves of the T_3_ and T_4_ transgenic lines by 2 × Taq Plus Master Mix (Vazyme, Nanjing, China). The PCR was performed with 10 μM final concentrations of primers and the following program: 94 °C for 5 min; 35 cycles at 94 °C for 30 s, 54 °C for 1 min and 72 °C for 2 min; and 72 °C for 5 min for the final extension [76]. The specific identification primers (Additional file S2: Table S2) used in PCR amplification were designed according to the vector and the *GsABCI1* sense fragment sequence.

### 4.5. Phenotypes of GsABCI1 Transgenic Lines

To analyze the phenotype of Al stress-related genes in wild type (WT) and *GsABCI1* overexpression lines, the seeds of T_3_ homozygous transgenic soybean lines and WT were germinated and adapted in a 0.5 μM CaCl_2_ hydroponic solution (pH = 5.8) for one day. The identified seedlings of soybean were transferred into a 0.5 μM CaCl_2_ hydroponic solution (pH 4.5) containing 0, 50, 100, or 150 μM AlCl_3_ for 12 h. Like the previous method, the *A. thaliana* seeds of T_3_ transgenic lines and WT were sterilized by 0.1% HgCl_2_ solution and grown on 1/2 mannitol salt (MS) agar plates (2% sucrose, 0.8% agar, pH 5.8) [75]. To break dormancy and ensure uniform germination, the *A. thaliana* seeds were initially cultured on MS medium for 2 days in the dark at 4 °C. Then, the uniform (about 1 cm) *A. thaliana* seedlings were moved to 1/2 MS agar plates (2% sucrose, 0.8% agar, pH 4.5) with 0, 25, 50, 75, 100, or 150 μM AlCl_3_ for two days [76].

In the phenotypic experiment, plants were grown in the greenhouse under the following conditions: 21–23 °C, 60% relative humidity, 100 μmol m^−2^ s^−1^ light intensity, and 16 h light/8 h dark cycles. The seedlings were photographed and labeled in sequence before and after Al^3+^ treatment, and then the length of the main root was analyzed using ImageJ. Al^3+^ sensitivity was evaluated by relative root elongation expressed as (root elongation with Al^3+^ treatment/root elongation without Al) × 100% [78].

In addition, the soybean root systems of treated plants were scanned by an Epson Expression 10000 XL scanner (Epson, Japan) to determine the relative total area, relative total root length, and relative total volume to assist in the evaluation of Al^3+^ tolerance [79]. All experiments were repeated at least three times with 16 biological replicates.

### 4.6. Bioinformatics Analysis of GsABCI1

Vector NTI was used for the amino acid sequence alignments of *GsABCI1* with the ABC transport system permease protein (ABC.X2.P) genes in *O. sativa* (*OsSTAR2, Os05g02750*), *A. thaliana* (*ALS3, At2G37330*), *G. max* (*GmABCI13, Gm10g047100*), *P. vulgaris* (*Phvul001g172900*), and *V. unguiculata* (*Vigun01g155100*). The phylogenetic tree analysis using MEGA-X was based on the JTT model and was enhanced by iTOLs (https://itol.embl.de/ (accessed on 6 June 2021)). Peptide sequence information was obtained from Phytozome (http://phytozome.jgi.doe.gov/pz/portal.html (accessed on 6 June 2021)). The transmembrane domain prediction was performed by SMART (http://smart.embl-heidelberg.de/ (accessed on 6 June 2021)). The protein model is predicted by Protein Homology/nalogy Recognition Engine v2.0 (Phyre2, http://www.sbg.bio.ic.ac.uk/ (accessed on 6 June 2021)).

### 4.7. Subcellular Localization of GsABCI1

To analyze the subcellular localization of *GsABCI1* protein*,* the *GsABCI1* (lacking a termination codon) gene was inserted at the 5′-terminus of the GFP gene in the pCMBIA1302-GFP vector under the control of the 35S promoter to express a GsABCI1-GFP fusion protein in *N. benthamiana* cells. The recombinant vector pCMBIA1302-*GsABCI1* was then transformed into the *A. tumefaciens* strain GV3101 by the freeze-thaw method and confirmed by molecular identification. The coding region of *GsABCI1* was amplified using gene-specific primers (Additional file S2: Table S2; the *NcoI* and *Spel* sites are underlined). The transient expression of the GsABCI1-GFP fusion protein was observed using a Lecia TCS SP8 STED 3× confocal laser scanning microscope (Lecia, Solms, Germany) [80].

### 4.8. Expression Pattern of GsABCI1

In the experiment, to investigate the expression pattern of *GsABCI1* in wild soybeans (*G. soja*), the plants were subjected to the following treatments: to determine time-dependent expression, plants were treated with a solution containing 50 μM AlCl_3_ for 0, 2, 4, 8, 12, and 24 h; to determine dose-dependent expression, plants were exposed to a solution containing 0, 5, 10, 25, 50, or 100 μM AlCl_3_ for 8 h; to determine tissue-specific expression, seedling plants were cultivated in a solution with 0 or 50 μM AlCl_3_ for 12 h; and to determine expression in response to metals and different pH values, seedling plants were grown under different pH conditions or in solutions (pH 4.5) containing 50 μM AlCl_3_, 23 μM CdCl_3_, 10 μM LaCl_3_, or 0.5 μM CuCl_2_.

To investigate the expression pattern of *GsABCI1*, total RNA was extracted using TRNzol Reagent (Tiangen Biotech, Beijing, China), and the conversion to cDNA was performed by HiScript^®^ III RT SuperMix for qPCR (+gDNA wiper) (Vazyme, Nanjing, China) according to the manufacturer’s procedures. The cDNA quality was assessed by PCR using specific primers for ACTIN3 to exclude genomic DNA contamination. The primers for qRT-PCR were designed using Primer Premier 6.0 software. qRT-PCR was performed in the 96-well (20 μL) format using the ChamQ™ Universal SYBR^®^ qPCR Master Mix (Vazyme, Nanjing, China) for Real-Time PCR on a CFX96™ Touch Real-Time PCR System (Bio-Rad, Hercules, CA, USA), which included three technical replicates. ACTIN3 was used as an internal control. The expression levels of all candidate genes were determined by using the 2^−ΔΔct^ method [70,79]. All primers used in qRT-PCR are shown in Additional file S2: Table S2.

### 4.9. Analysis of Al Accumulation Patterns

To analyze Al accumulation patterns, 5-day-old WT and transgenic soybean seedlings were exposed to a CaCl_2_ hydroponics solution (pH 4.5) with 25 μM AlCl_3_ for 24 h. Subsequently, to evaluate the amount of surface-bound Al, the roots (0–2 cm) were stained with hematoxylin for 30 min, washed three times with ddH_2_O, and observed using a physical microscope (Leica Microsystems, Leica, Germany) [81]. To quantify the Al status of roots, the seedlings were exposed to a CaCl_2_ hydroponics solution (pH 4.5) with 0, 10, 25, or 50 μM AlCl_3_ for 24 h. Then, the roots (0–6 cm, 10 roots, three replicates) were digested, and the Al^3+^ was extracted by 2_N_ HCl for 24 h with occasional shaking. The Al^3+^ concentration in the extracts was determined by inductively coupled plasma-atomic emission spectrometry (ELEMENT™ Series ICP-MS optical emission spectrometer, Thermofisher, Waltham, MA, USA) [38].

### 4.10. Root Component Analysis of GsABCI1 Transgenic Lines

We selected the soybean roots (0–2 cm) treated with 0, 50 μM AlCl_3_ in Section 4.9 for root component analysis. The DNS colorimetric method was used for hemicellulose measurement. In short, hemicellulose is converted into reducing sugar after acid treatment, which reacts with DNS to generate a red-brown substance. The product has a characteristic absorption peak at 540 nm, and the content of hemicellulose can be quantitatively detected by changing the absorbance value [37]. Pectin aldose was measured by the carbazole colorimetric method. The basic principles were as follows: protopectin was hydrolyzed into soluble pectin in dilute acid solution, and further converted into galacturonic acid; galacturonic acid condensed with carbazole in a strong acid environment to generate a purplish red compound; the product had a characteristic absorption peak at 530 nm; the content of protopectin could be calculated by the change of the absorption value [82].

## 5. Conclusions

In summary, a half-size ABC transporter gene, *GsABCI1*, was isolated and its aluminum-induced expression pattern was described in this study. These results showed that *GsABCI1* was responsive to aluminum stress and might enhance aluminum tolerance through aluminum transport or indirectly changing root components. Those results will provide some information for understanding the gene function of the ABC family and soybean germplasm development.

## Data Availability

Not applicable.

## Supplementary Materials

The following are available online at https://www.mdpi.com/article/10.3390/ijms222413264/s1.
