# Supplementary material for: Identification of an ATP-Binding Cassette Transporter Implicated in Aluminum Tolerance in Wild Soybean (Glycine soja)
Source: Int J Mol Sci. 2021 Dec 9;22(24):13264. doi: 10.3390/ijms222413264 (PMC8706246; doi:10.3390/ijms222413264)
Supplement: Supplementary file 1 [file ijms-22-13264-s001.zip › Additional file S1 Table S1ú║CDS of GsABCI1.pdf]

Table S1. CDS sequencing information of *GsABCI1*.

1	ATGGAATCTA ATTTCTTCTT TAAAACCAAT ATGCTAACTC TGAACCACAC ATTGCAGCAT
61	GAATTAGTGG TCCCTTTAGT TGGCACGGAT TGGTCATGGA TGATGGAGTT CCTGAAGGGT
121	ATGGTGAAGC CAGTGGCTGC CACTGCAGTG GTGTGTTTGG CTGTGGCCTT GTCCTTCTAC
181	CAGAAGCTGG GGCTGGAGCT AGAGATGGTT GTTGCCATTG TGAGGGCATT TATTCAACTT
241	TCTATCATTG GCTTTGTGTT GGAGTTCATT TTCCGCCAGG ACAACGCTGG ATGGATCCTC
301	CTAGCATACC TTTTCATGGT ATCAATAGCG GGTTACACTG CGGGTCAGCG TGCGAAACAT
361	GTTCCTCGTG GGAAGTATGT GGCAGGGGCC TCCATTCTCA CAGGAACTGC AGTGACCATG
421	TTGGTGCTGG TAGCTTTGAG CGTGTTCCCT TTCACTCCTA GATATATCAT ACCTGTGGCT
481	GGCATGATGG TTGGGAACTC AATGACAGTT ACCGGTGTTA CCATGAAAAG ACTCAGAGAC
541	GACATCAAAA CTCAAATGAA CTTGGTAGAG ACAGCATTGT CTCTTGGTGC AACCCCACGA
601	CAAGCAACGC ATGAACAAGT GAAAAGGGCT CTGATATTGG CCCTTTCTCC GGTGGTGGAC
661	AACACCAAAA CAGTGGGTCT AATATCCCTT CCTGGGGCAA TGACTGGTCT CATTATGGGA
721	GGGGCATCGC CGTTGGAAGC AATTCAACTA CAGATTGTGG TGATGAATAT GATGATTGGT
781	GCAGCAACTG TTAGCAGCAT AATGGCTACA TACCTCTGTT GGCCAGCATT CTTCACCAAG
841	GCCTACCAAT TGGAAACCAA AGTCTTCTCA AGTTGA
