# Supplementary material for: Identification of an ATP-Binding Cassette Transporter Implicated in Aluminum Tolerance in Wild Soybean (Glycine soja)
Source: Int J Mol Sci. 2021 Dec 9;22(24):13264. doi: 10.3390/ijms222413264 (PMC8706246; doi:10.3390/ijms222413264)
Supplement: Supplementary file 1 [file ijms-22-13264-s001.zip › Additional file S2ú║Table S2 Primers used in the study..pdf]

Table S2. Primers used in the study.

Serial Number	Primer Name	Primer Sequence (5′ to 3′)	Purpose
1	Clone-*GsABCI1*-F	ACACATTCTGGGGAAGGTCG	*GsABCI1* Cloning
2	Clone-*GsABCI1*-R	TCACCGATTCAAGTTAGGTGG	
3	pTF101.1-*GsABCI1*-F	GCTCTAGA ACACATTCTGGGGAAGGTCG	*GsABCI1*-pTF101.1
4	pTF101.1-*GsABCI1*-R	CGAGCTC TCACCGATTCAAGTTAGGTGG	
5	Detect-F	ACTTCTACACAGCCATCGGTCC	Molecular identification
6	Detect-R	TCACCGATTCAAGTTAGGTGG	
7	GFP-*GsABCI1*-F	GGATCTTCCAGAGATCCATGGCGATGGAATCTAATTTCTTCTTTAAAACCA	*GsABCI1*-pCMBIA1302
8	GFP-*GsABCI1*-R	CTGCCGTTCGACGATACTAGTAACTTGAGAAGACTTTGGTTTCCAA	
9	Actin-F	GCACCACCGGAGAGAAAATA	qRT-PCR
10	Actin-R	GTGCACAATTGATGGACCAG	
11	q*GsABCI1*- F	ACGACAAGCAACGCATGAAC	
12	q*GsABCI1*- R	TTGAATTGCTTCCAACGGCG	

All the primers were used for gene cloning, vector construction, molecular identification, and expression analysis.
