# Supplementary material for: Identification of an ATP-Binding Cassette Transporter Implicated in Aluminum Tolerance in Wild Soybean (Glycine soja)
Source: Int J Mol Sci. 2021 Dec 9;22(24):13264. doi: 10.3390/ijms222413264 (PMC8706246; doi:10.3390/ijms222413264)
Supplement: Supplementary file 1 [file ijms-22-13264-s001.zip › Additional file S3ú║Figure S1 .pdf]

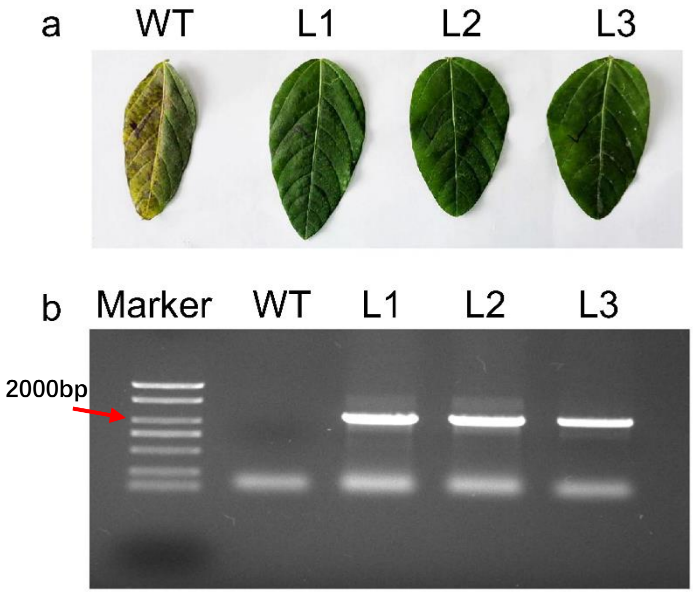

Figure S1. Detection of *GsABCI1* transgenic lines (**a**) Herbicides identification. The first terately compound leafs of soybeans were sprayed with herbicides Liberty ®. Observing leaf wilting after three days. (**b**) Molecular identification of *GsABCI1* transgenic soybean. Marker = 5000 bp (Stripe size from top to bottom 5000, 3000, 2000, 1000, 750, 250, 100, separately).
