# Supplementary material for: Identification of an ATP-Binding Cassette Transporter Implicated in Aluminum Tolerance in Wild Soybean (Glycine soja)
Source: Int J Mol Sci. 2021 Dec 9;22(24):13264. doi: 10.3390/ijms222413264 (PMC8706246; doi:10.3390/ijms222413264)
Supplement: Supplementary file 1 [file ijms-22-13264-s001.zip › Additional file S4ú║Figure S2 .pdf]

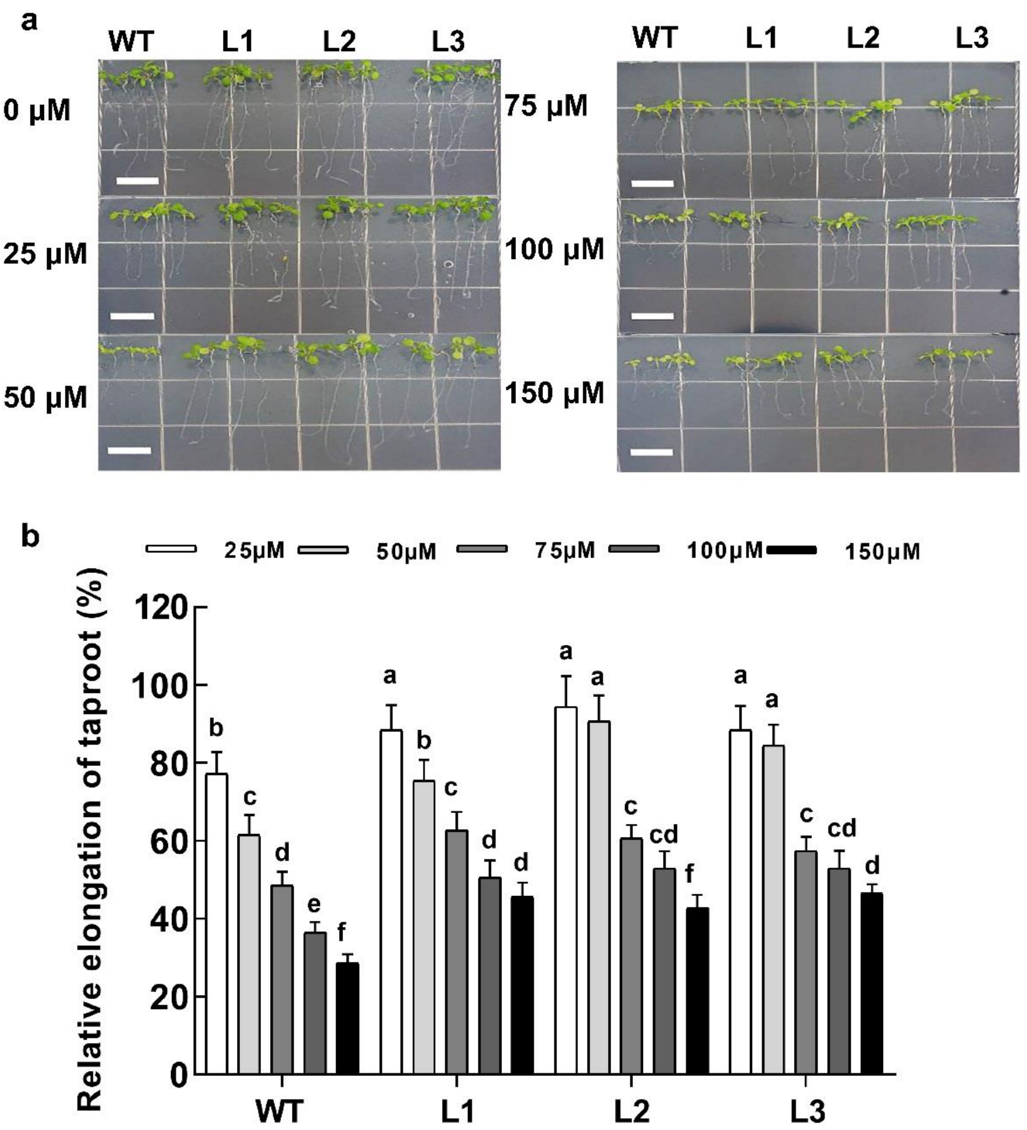

Figure S2：Phenotypic analysis of *GsABCI1* transgenic *Arabidopsis* lines. (**a**)

Root morphology analysis of WT and three transgenic lines. Seeding was transferred into 1/2 MS agar plates with different Al treatment for 2 days. Bar = 1 cm. (**b**) Taproot relative elongation analysis of WT and three transgenic lines. Root length was measured by ImageJ software before and after Al treatment. Data are means ± SD (n = 12). Different letters indicate significant differences at $p < 0.05$ by Student's *t*-test.
