# Supplementary material for: Identification of an ATP-Binding Cassette Transporter Implicated in Aluminum Tolerance in Wild Soybean (Glycine soja)
Source: Int J Mol Sci. 2021 Dec 9;22(24):13264. doi: 10.3390/ijms222413264 (PMC8706246; doi:10.3390/ijms222413264)
Supplement: Supplementary file 1 [file ijms-22-13264-s001.zip › Additional file S5ú║Table S3 Medium used in the generation of GsALS3 transgenic soybean..pdf]

Table S3. Medium used in the generation of *GsALS3* transgenic soybean.

Serial Number	Medium Name	Medium Composition
1	Germination medium（GM）	10 % B5 primary elements, 10 % B5 trace elements, 2 % Sucrose, 1 % Fe-EDTA,0.3 % agar, 0.1 % B5 vitamins, pH 5.8.
2	Cocultivation media (CM)	10 % B5 primary elements, 10 % B5 trace elements, 2% Sucrose, 1 % Fe-EDTA , 0.1 % B5 vitamins, 3.9 g/L 2-[N-morpholino] ethanesulfonic acid (MES), 0.25 mg/L gibberellic acid (GA_3), 1.67 mg/L N-6-benzylaminopurine (BAP), 200 μm/L Acetosyringone (As), 400 mg/L L-cysteine, 155 mg/L Dithiothreitol (DTT) and 0.5% Agar, pH 5.4.
3	Sprout induce media (SIM)	10 % B5 primary elements, 10 % B5 trace elements, 2% Sucrose, 1 % Fe-EDTA , 0.1 % B5 vitamins, 5.9 g /L 2-[N-morpholino] ethanesulfonic acid (MES), 1.67 mg/L 6-BA, 250 mg/L Cefotaxime sodium, 5 mg/L Phosphinothricin, 200 mg/L Timentin, 0.5% Agar, pH 5.7.
4	Sprout elongation media (SEM)	10 % M5 primary elements, 10 % M5 trace elements, 2% sucrose, 1 % Fe-EDTA , 0.1 % M5 vitamins, 5.9 g/L 2-[N-morpholino] ethanesulfonic acid (MES), 5 mg/L Phosphinothricin, 10 mg/L Asparagine, 10 mg/L Glutamine, 1 mg/L IAA, 1 mg/L GA_3, 1 mg/L Zeatin, 200 mg/L Timentin, 250 mg/L Cefotaxime sodium and 0.5% Agar, pH 5.7.
5	Root spread media (RSM)	10 % M5 primary elements, 10 % M5 trace elements, 2% Sucrose, 1 % Fe-EDTA , 0.1 % M5 vitamins, 6.0 g/L 2-[N-morpholino] ethanesulfonic acid (MES), 10 mg/L Asparagine, 10 mg/L Glutamine, 200 mg/L Timentin, 250 mg/L Cefotaxime sodium, 0.5% agar, pH 5.6.
6	B5 primary elements (20×)	KNO_3 2500 mg/L, $CaCl_2$ 150 mg/L, $MgSO_4 \cdot 7H_2O$ 250 mg/L, $(NH_4)_2SO_4$ 134 mg/L, $NaH_2PO_4 \cdot H_2O$ 150 mg/L.
7	B5 trace elements (200×)	H_3BO_3 3 mg/L, $MnSO_4 \cdot 4H2O$ 10 mg/L, $ZnSO_4 \cdot 7H_2O$ 2 mg/L, KI 0.75 mg/L, $Na_2MoO_4 \cdot 2H_2O$ 0.25 mg/L, $CuSO_4 \cdot 5H_2O$ 0.025 mg/L, $CoCl_2 \cdot 6H_2O$ 0.025mg/L.
8	B5 vitamins (100×)	Myo-inositol 10 mg/L, VB_1 1 mg/L, VB_6 0.1 mg/L, Nicotinic Acid 0.1 mg/L.
9	M5 primary elements (20×)	NH_4NO_3 1650 mg/L, KNO_3 190 mg/L, $CaCl_2$ 440 mg/L, $MgSO_4 \cdot 7H_2O$ 370 mg/L, $KH_2PO_4 \cdot H_2O$ 170 mg/L.
10	M5 trace elements (200×)	H_3BO_3 6.2 mg/L, $MnSO_4 \cdot 4H_2O$ 22.3 mg/L, $ZnSO_4 \cdot 7H_2O$ 8.6 mg/L, KI 0.83 mg/L, $Na_2MoO_4 \cdot 2H_2O$ 0.25 mg/L, $CuSO_4 \cdot 5H_2O$ 0.025 mg/L, $CoCl_2 \cdot 6H_2O$ 0.025mg/L.
11	M5 vitamins (100×)	Glycine 2 mg/L, VB_1 1mg/L, VB_6 0.5 mg/L, Nicotinic Acid 0.5 mg/L.
